# Functional Genomics, Genetics, and Bioinformatics

**DOI:** 10.1155/2015/184824

**Published:** 2015-04-22

**Authors:** Youping Deng, Hongwei Wang, Ryuji Hamamoto, David Schaffer, Shiwei Duan

**Affiliations:** ^1^Department of Internal Medicine, Rush University Cancer Center, Rush University Medical Center, Chicago, IL 60612, USA; ^2^Department of Medicine, University of Chicago, Chicago, IL 60637, USA; ^3^Department of Bioengineering, Binghamton University, Binghamton, NY 13902, USA; ^4^School of Medicine, Ningbo University, Ningbo, Zhejiang 315211, China

Biology has become the land of the “-omics,” including genomics [1], transcriptomics [2, 3], epigenomics [4], proteomics [5], lipidomics [6, 7], and metabolomics [8]. Each of these “-omics” generates a huge amount of high-throughput data, and it is a challenge both to analyze these data and to further investigate the function of specific molecules. Though more genomes have been completed due to the rapid development of sequencing technology [9], we cannot understand the information contained within a genome until we mine out its implicated functions including downstream transcription, translation, epigenetics modulation, and metabolic pathways. In this special issue, we mainly focus on functional “-omics” and bioinformatics.

The Peer-reviewed papers are collected in the special issue. They are approximately divided into three areas: bioinformatics, functional genomics, and functional genetics. The majority of the papers are purely bioinformatics related papers. We define bioinformatics papers as those using computational tools or developing methods to analyze functional “-omics” data without using wet labs. Two papers fell into the category of functional genomics, which is focused on using whole genome level wet-lab technology to find important molecules and investigate their potential functions. Five papers are considered as functional genetics papers. Functional genetics is a broad concept here and these papers are concentrated on studying the molecular functions and mechanisms of individual molecules using wet-lab experimental approaches.

*Bioinformatics*. In the bioinformatics papers, four papers deal with transcriptomics data. F. Wang et al. developed a novel approach for coexpression analysis of E2F1-3 and MYC target genes in chronic myelogenous leukemia (CML); they found a significant difference in the coexpression patterns of those candidate target genes between the normal and the CML groups. It is challenging to analyze the quantity of image data on gene expression. A. Shlemov et al. developed a method called 2D singular spectrum analysis (2D-SSA) for application to 2D and 3D datasets of embryo images related to gene expression; it turned out to work pretty well. J. Li et al. characterized putative* cis*-regulatory elements (CREs) associated with male meiocyte-expressed genes using* in silico* tools. They found that the upstream regions (1 kb) of the top 50 genes preferentially expressed in* Arabidopsis* meiocytes possessed conserved motifs, which were potential binding sites of transcription factors. NAGNAG alternative splicing plays an important role in biological processes and represents a highly adaptable system for posttranslational regulation of gene function. Interestingly, X. Sun et al. identified about 31 NAGNAG alternative splicing sites that were identified in human large intergenic noncoding RNAs (lincRNAs).

Three papers are focused on the deification of new gene family members and gene evolution. Conotoxins are small disulfide-rich neurotoxic peptides, which can bind to ion channels with very high specificity and regulate their activities. H. Ding et al. developed a novel method called iCTX-Type, which is a sequence-based predictor that can be used to identify the types of conotoxins in targeting ion channels. A user-friendly web tool is also available. Y.-Z. Zhou et al. analyzed the evolution pattern and function diversity of PPAR gene family members based on 63 homology sequences of PPAR genes from 31 species. They found that gene duplication events, selection pressures on HOLI domain, and the variants on promoter and 3′UTR are critical for PPARs evolution and acquiring diversity functions. There has recently been considerable focus on its two human pathogenic species* N. meningitidis* and* N. gonorrhoeae*, which belong to* Neisseria*, a genus of gram-negative bacteria. D. Yu et al. selected 18* Neisseria* genomes, preformed a comparative genome analysis, and identified 635 genes with recombination signals and 10 genes that showed significant evidence of positive selection. Further functional analyses revealed that no functional bias was found in the recombined genes. The data help us to understand the adaptive evolution in* Neisseria*.

One paper tried to solve the key algorithm issue called the all-pairs suffix-prefix matching problem, which is crucial for de novo genome assembly. M. H. Rachid et al. developed a space-economical solution to the problem using the generalized Sadakane compressed suffix tree. One paper conducted a comparative genomics analysis. R. Cecagno et al. found that the versatile gene repertoire in the genome of rhizosphere bacterium* Azospirillum amazonense* could have been acquired from distantly related bacteria from horizontal transfer. They also demonstrated that the coding sequence related to production of phytohormones, such as flavin monooxygenase and aldehyde oxidase, is likely to represent the tryptophan-dependent TAM pathway for auxin production in this bacterium. They conclude that the genomic structure of the bacteria has evolved to meet the requirement for adaptation to the rhizosphere and interaction with host plants.

One article conducted a meta-analysis. H. Ye et al. have demonstrated that rs2228671 is a protective factor of CHD in Europeans. One paper is concentrated on the microorganism bioinformatics. Y. Ding et al. recognized the roles of the synonymous codon usage in the formation of nsp1*α* structure of porcine reproductive and respiratory syndrome virus PRRSV.

*Functional Genomics*. There are two papers that conducted gene association studies based on genome wide data. J. Li et al. found that the presence of ATT*ε*4haplotype was associated with an increased risk of mental retardation (MR) in children but did not find any significant association between single loci of the four common ApoE polymorphisms (−491A/T, −427T/C, −219T/G, and *ε*2/3/4) and MR or borderline MR. J. Zhou et al. did not find an association between rs7529229 and chronic heart disease (CHD) in Han Chinese. However, their meta-analyses indicated that rs7529229 was associated with the CHD risk in Europeans.

*Functional Genetics*. There are 5 articles that investigate the individual gene function in different areas. Two papers are related to neural diseases. G.-M. Chang et al. found that activating NF-*κ*B signaling pathway can protect intestinal epithelial cell No. 6 against fission neutron irradiation. X.-S. Liu et al. demonstrated that hepatocyte growth factor (HGF) could promote the regeneration of damaged Parkinson's disease (PD) cells at higher efficacy than the supernatant from hUC-MSCs alone. Thus, the combination of hUC-MSC with HGF could potentially be a new biological treatment for PD. One paper is focused on cancer. N. Ji et al. found that celastrol had antiprostate cancer effects partially through the downregulation of the expression level of hERG channel in DU145 cells, suggesting that celastrol may be a potential agent against prostate cancer with a mechanism of blocking the hERG channel. One paper is studying heart disease. Z. Lu et al. reported that the levels of NT-proBNP and CCR were closely related to the occurrence of HF and were independent risk factors for heart failure (HF). Meanwhile, there was a significant negative correlation between the levels of NT-proBNP and CCR. One interesting paper is trying to understand the function of Japanese encephalitis virus (JEV), and they have demonstrated that RNA recombination in JEV occurs unequally in different cell types. They conclude that the adjustment of viral RNA to an appropriately lower level in mosquito cells prevents overgrowth of the virus and is beneficial for cells to survive the infection.

In summary, this special issue presents a broad range of topics from functional genomics, genetics, and bioinformatics. It covers a variety of diseases such as cancer, heart, and neural and infectious diseases. The study organisms include human, mouse, plant, and microorganisms. We hope that the readers will find interesting knowledge and methods in the issue.

*Youping Deng* *Hongwei Wang* *Ryuji Hamamoto* *David Schaffer* *Shiwei Duan*
